# Birth Advantages in Male Italian Soccer: How They Influence Players Youth Career and Their Future Career Status

**DOI:** 10.3390/sports12040103

**Published:** 2024-04-09

**Authors:** Gabriele Morganti, Paolo Riccardo Brustio, Bruno Ruscello, Gennaro Apollaro, Elvira Padua, Adam L. Kelly

**Affiliations:** 1 Department of Human Sciences and Promotion of the Quality of Life, San Raffaele Roma Open University, 00166 Rome, Italy; gabriele.morganti@uniroma5.it (G.M.); bruno.ruscello@uniroma5.it (B.R.); elvira.padua@uniroma5.it (E.P.); 2 Department of Clinical and Biological Sciences, University of Turin, 10126 Turin, Italy; paoloriccardo.brustio@unito.it; 3 Department of Industrial Engineering, Faculty of Engineering, “Tor Vergata” University, 00133 Rome, Italy; 4 LUISS SportLab, 00197 Rome, Italy; 5 Department of Neuroscience, Rehabilitation, Ophthalmology, Genetics and Maternal Child Health, University of Genoa, 16126 Genoa, Italy; gennaro.apollaro@edu.unige.it; 6 Research for Athlete and Youth Sport Development (RAYSD) Lab, Centre for Life and Sport Sciences (CLaSS), Faculty of Health, Education and Life Sciences, Birmingham City University, Birmingham B15 3TN, UK

**Keywords:** birth advantages, relative age effects, birthplace effects, talent identification, talent development

## Abstract

Soccer organizations generally adopt deterministic models within their talent pathways. In this framework, early ability and results are emphasized, leading to selection biases, such as birth advantages (i.e., relative age effects and birthplace effects), which research has shown affect both early developmental experiences and continued sporting involvement. Accordingly, this study aimed to (a) provide further test of birth advantages in Italian youth soccer by exploring the birth quarter (BQ) and birthplace (BP) distribution of 1050 male Italian players born between 1999 and 2001 who competed in the national U17 championship throughout the 2015–16 season and (b) investigate how birth advantages influenced selected players’ future career status. Chi-square goodness-of-fit tests revealed early born players, and players born in North Italy were overrepresented at the youth level (*p*-values < 0.0001). Successive prospective analysis revealed only 18% of players developed into professional-level soccer players. Chi-square tests of independence indicated that players’ BP was associated with their future career status (*p* < 0.0001), whereas their BQ was not (*p* = 0.459). Odds ratios showed players born in North Italy were five times more likely to complete the youth-to-senior transition than those born in South Italy. These findings highlighted environmental factors influence Italian players’ early developmental experiences and their future career status.

## 1. Introduction

Youth soccer organizations and governing bodies often follow a deterministic approach to Talent Identification and Development (TID; see Morganti et al. [1] for a discussion). Deterministic TID are based on identifying the most talented players in the early stages of their development to support their sporting journey by selecting them into optimal learning environments (e.g., competent coaches and technical staff, appropriate training structures, and higher competition levels) [2,3]. Early entry into such high-performance settings and learning environments, where players can accumulate hours of deliberate practice in soccer, is viewed as the beginning of the sporting “profession” whilst also considered a prerequisite for future soccer success [4]. Indeed, early identification, and consequent early specialization of the selected few, are considered two of the basic tenets of the Standard Model of Talent Development (SMTD) [5]. The SMTD is a pyramidical-shaped, progressive model of athlete selection and development that focuses all its efforts on achieving eventual performance. Players’ progression through the model (i.e., step by step until an individual reaches the top of the pyramid) is based on possessing the required traits to fit in with the organization and includes the marginalization and exclusion of most individuals [6], who do not show high performance levels and early ability, as well as character and psychological characteristics aligned with the culture of reference [7]. As such, the focus on players’ eventual performance is overshadowed by the concept of achievement now [8]. This indicates possible hypocrisy in youth soccer, whereby soccer organizations remark on their long-term developmental focus (i.e., eventual performance) but, at the same time, during the identification processes, are mostly concerned with trying to find the best possible youth players (8–16 years of age, i.e., actual performance) [9].

During TID processes, athletes with the potential to succeed are selected, while the others are deselected. In soccer, it is considered possible to identify and select talented players early (e.g., aged 6, 10, or 13 years), using their current ability and evaluations of in-match performance as benchmarks [10]. This emphasizes the need for early sport-specific engagement, whereby “elite” youth athletes (throughout the paper, the word elite, when used in association to a youth athlete, will be put in quotation marks to shine a light on the fallacy of such a term, often used by governing bodies and academics alike in reference to youth sport, which only reinforces concepts of determinism and child athlete professionalization [11]), specialize in a single sport and are demanded to follow rigid schedules (e.g., attend daily training sessions and competitive fixtures every week), being expected to sacrifice their social [12] and educational spheres of life [13]. However, research conducted in this area has shown how the reality of selection decisions are difficult tasks to uptake whilst also considering the surrounding uncertainty and nonlinearity around athletes’ developmental processes [14,15]. Despite this early and full engagement in a single sport, most “elite” youth athletes are not able to maintain their “elite” status once they grow older and reach adulthood [16,17,18,19]. As an example, a recent meta-analysis conducted by Güllich et al. [20], revealed how early recruitment negatively correlates with senior world class performance, both in individual and team sports. In German soccer, once selected for an academy, young players had >70% chance of being deselected in the next five years, with only 7% of players progressing from U10 to U19 [21], while the 90% of U17 youth soccer players involved in these academies are unable to sign a professional contract later in their career [22]. As such, contrary to expectations, players recruited at an earlier age by professional club academies do not show a higher probability of attaining a professional contract at a senior level compared to peers who entered the talent pathway at a later age [23]. In line with this, Boccia et al. [24] investigated the youth-to-senior transition across the Italian national soccer representatives and found that less than 10% of players from the U16 teams were able to complete the transition and that 40% of players selected for the U21 were subsequently selected for the senior national team. Therefore, it is only as players get older (i.e., ≥21 years) that their performance at the youth level can be correlated with their performance at the senior level.

Research has shown how selecting players based on their actual level of performance often leads to several selection biases [25,26]. Birth advantages (i.e., relative age effects (RAEs) and birthplace effects) have been defined by Hancock and Côté [27] as “variables present at birth that have long-lasting influences throughout development” and are the most studied bias affecting all TID across various sports [28] (p. 16). RAEs arise from the decision of youth sports organizations to adopt a cut-off criterion that groups children into(bi)annual age groups. Past studies have presented how such a strategy favours the selection of players born at the begin of the selection year (1 January in Italy) compared to those born at the end of the selection year (31 December in Italy) [29]. Due to their chronological age, relatively older players hold the advantage of greater performance capabilities and are therefore preferred, by a results-driven system, over their younger peers [30]. Research has already shown how the Italian soccer landscape suffers from RAEs over time [31].

Birthplace effects are related to the community’s physical aspect (i.e., proximity and access to facilities, open spaces, green areas, and the presence of sports organizations and youth clubs) [32] and its socioeconomic, cultural, political, and historical characteristics [33,34,35,36]. These factors influence children’s early developmental experiences in sport and thus contribute to early skill development [37]. As an example, Morganti et al. [38] recently demonstrated how well-known regional disparities in Italy influence soccer players’ developmental pathways. More in detail, the authors suggested how the Italian North–South divide, often labelled with the expression “southern question”, which past research has attributed to a path dependency towards a clientelist style of politics (i.e., corruption and poor investments) [39,40], interacts with Italian players’ likelihood of representing Italy internationally both at the youth and senior levels, favouring players born in the north and central regions.

Research conducted on birth advantages has investigated how relative age may impact athletes’ youth-to-senior transition [41,42]. Studies have demonstrated how, both in English and Italian soccer, selected later-born players have an increased likelihood of completing the transition [43,44]. In contrast, very few studies have investigated the implications of the birthplace in the transition to the professional level. Brown et al. [45] showed in English and Welsh cricket that white British players who were privately educated were 34 times more likely to achieve a professional status compared to British South Asian players who were state educated, thus demonstrating how both relative access to wealth and ethnicity impact athlete developmental experiences and future career outcomes in sports.

Accordingly, this study aims to (a) provide a further test of birth advantages in Italian youth soccer, investigating their influences on youth players’ likelihood of being selected into a high-performance environment, and (b) explore how birth advantages interact with selected players’ future career status. For this reason, this study was divided into two parts: Part 1 explored the birthdates and birthplaces of Italian youth soccer players who have played at the U17 Italian national level competition; Part 2 recorded and prospectively investigated the future career trajectories of these players to explore both how many were able to develop into professional level players and whether birth advantages influenced the youth-to-senior transition. For Part 1 of the study, it was hypothesized that RAEs and birthplace effects were both largely present at youth levels. For Part 2 of the study, it was hypothesized that the majority of players included in this study were not able to become professional soccer players and that their youth-to-senior transition was associated with both relative age and birthplace.

## 2. Materials and Methods

### 2.1. Subjects

To be eligible for inclusion in this study, a player must have played in the U17 male Italian national soccer championship during the 2015–2016 season (n = 1290). Players not born in Italy and players whose birthplaces were not retrievable were omitted from the study (n = 240). After this initial screening, a total sample of 1050 Italian male soccer players born between 1999 and 2001 (both years included) were considered for the statistical analyses. Because all data were freely available from the internet, no approval by an ethical committee was required.

### 2.2. Procedures

**Figure 1:**
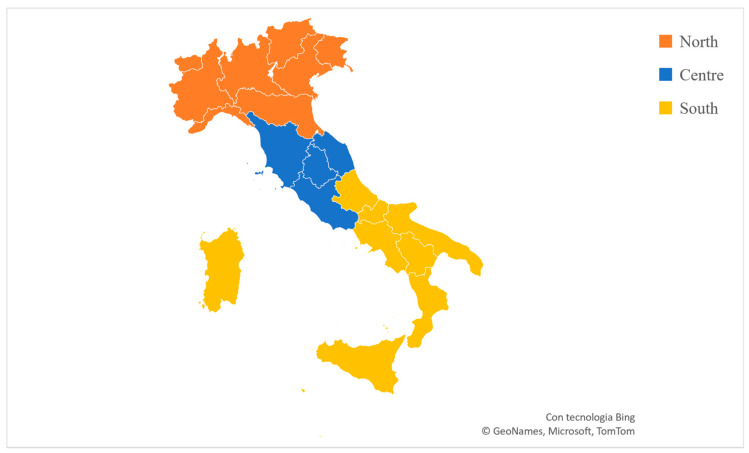
Italian map showing the microregions of Italy divided by macro-regions (North, Centre, and South).

The data for this study (i.e., players’ team selection, future career status, birthdates, and birthplace) were publicly available in the public domain and retrieved online from the Transfermarkt website (https://www.transfermarkt.it/campionato-under-17-a/startseite/wettbewerb/ITJ4/plus/?saison_id=2015) (accessed on 15 July 2023). Italy’s 20 microregions are divided into three macro-regions (i.e., North, Centre, and South; Figure 1). Players were classified based on their future career status on the date of data recording (July/August 2023) (Level 1 players: playing in the Italian or another top European soccer country first division; Level 2 players: playing in the Italian or another top European soccer country second or third divisions; Amateur players: playing at an amateur level; Former players: no more involvement in soccer organised practices); birthdate (Birth Quarter 1 (BQ1) = January, February, and March; BQ2 = April, May, and June; BQ3 = July, August, and September; and BQ4 = October, November, and December); and macro-region of birth (i.e., North, Centre, and South Italy). The observed birthdate and birthplace distribution of each cohort (i.e., youth and senior) were calculated for each BQ and macro-region and compared to the expected distribution, which was based on the general population norms that were obtained by census statistics [46,47].

### 2.3. Data Analysis

In Part 1 of this study, a chi-square goodness-of-fit test (χ2) was used to compare the observed U17 BQs and birthplace distribution to the general population norms distribution obtained from census statistics [46,47]. In Part 2 of this study, to explore the youth-to-senior transition, a prospective analysis was adopted, which consisted of calculating the transition rate as the frequency of players who achieved different future career status divided in Level 1, Level 2, Amateur, and Former players. The transition rates were calculated using a binomial proportion confidence interval (95% CI). Furthermore, a chi-square test for independence (χ2) was used to separately investigate both RAEs and birthplace (set as the independent variable) influence on future career status (set as the dependent variable).

Since chi-square statistics cannot reveal the magnitude and the direction of an existing relationship for significant chi-square outputs, the effect size (Cramer’s V) and odds ratios (ORs) were also calculated. The Cramer’s V was interpreted as follows: values between 0.05 and 0.09 indicated a weak effect size, 0.10–0.14 indicated a moderate effect size, 0.15–0.24 indicated a strong effect size, and 0.25 or more indicated a very strong effect size [48]. The ORs and 95% CIs were used to separately compare BQs and birthplaces for the achievement of youth and Level 1 status. These were calculated with the youngest group used as reference (BQ4), as well as for each macro-region (i.e., North vs. Centre; North vs. South; Centre vs. South). The ORs were calculated and interpreted following the procedures outlined by Szumilas [49], with CIs including 1 (i.e., 95% CI 0.90–1.10) marked no association. The results were considered significant for *p* < 0.05. Statistical analyses were conducted and maps of Italy were produced using Microsoft Excel (2019).

## 3. Results

**Figure 2:**
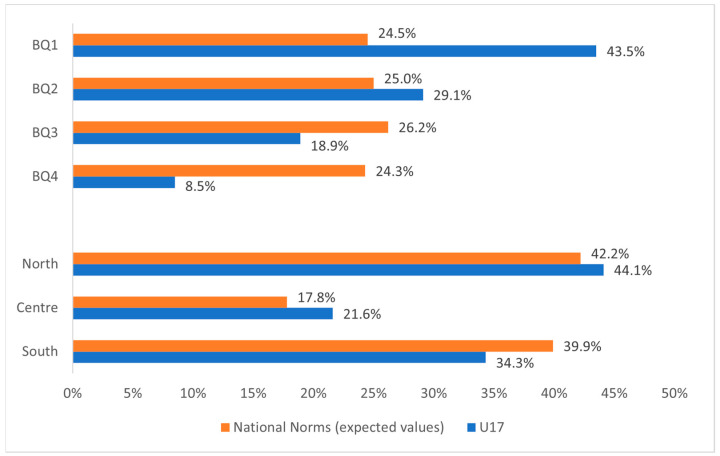
The observed BQs and birthplace distribution for the U17 Italian “elite” soccer players compared to the national norms (expected values).

The observed BQs and birthplace distribution for the U17 Italian “elite” soccer players, as well as the general population norms, are separately displayed in Figure 2. The results revealed that both the birthdate and birthplace distribution at the U17 level were significantly skewed when compared to their respective general population norms (birthdate: χ^2^ (3) = 281.848; *p* < 0.0001; strong effect size; birthplace: χ^2^ (2) = 17.718; *p* < 0.0001; very strong effect size). The descriptive ORs showed an increased likelihood of players born in BQ1, BQ2, and BQ3 of playing at the U17 “elite” level compared to players born in BQ4 (ORs BQ1 vs. BQ4 (95% CI) = 5.09 (3.83–6.78); BQ2 vs. BQ4 = 3.34 (2.50–4.84); BQ3 vs. BQ4 = 2.06 (1.52–2.79)). Moreover, the results showed an increased likelihood of players born in the north and in the centre of playing at the U17 “elite” level compared to players born in the south (ORs North vs. South (95% CI) = 1.22 (1.01–1.47); Centre vs. South = 1.41 (1.11–1.79)).

**Figure 3:**
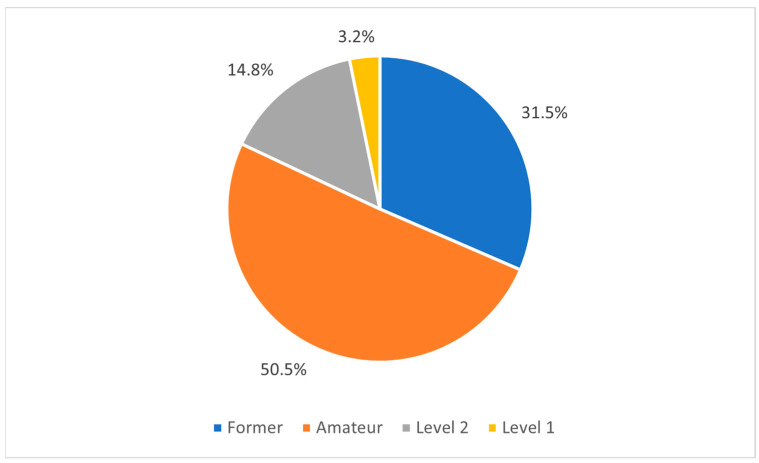
Future career status (prospective analysis) of former U17 “elite” Italian soccer players.

The prospective analysis showed that 96.8% (95.9–97.7) of Italian players who played at the U17 “elite” soccer level throughout the 2015-2016 season were not able to achieve the Level 1 player status once they grew older (Figure 3). More in detail, 3.2% (2.3–4.1) of players became Level 1 players and 14.8% (13–16.5) progressed as Level 2 players, while 50.5% (47.9–53) continued to play at the amateur level and 31.5% (21.2–34) were no longer involved in organized soccer.

Table 1 (birthdate) and Table 2 (birthplace) report the results from the chi-square test of independence. No association between players’ BQs and their future career status (*p* = 0.459; very strong effect size) were found, while, in contrast, the birthplace analysis revealed a statistically significant association between players’ birthplace and their future career status (*p* < 0.0001; moderate effect size; Level 1 players: North (expected value) = 73.5% (44%), Centre = 14.7% (21.7%), South = 11.7% (34.3%); Former players: North (expected values) = 31.4% (44.1%), Centre = 26% (21.6%), South = 42.6% (34.3%)).

In line with this, further statistical analyses using ORs highlighted how U17 “elite” Italian soccer players born in the north had the greatest likelihood of becoming Level 1 players later in their career compared to their compatriots born in the south (ORs North vs. South (95% CI) = 4.88 (1.32–17.95)).

## 4. Discussion

Soccer talent pathways generally follow a deterministic model of TID that emphasizes the promotion of early identification and specialization practices [5], which often leads to several selection biases (i.e., birth advantages) [50]. Accordingly, this study’s aim was twofold: (a) to provide a further test of birth advantages in Italian youth soccer and (b) to investigate whether birth advantages influenced selected players’ future career status. The results from Part 1 of this study revealed how the population of U17 “elite” Italian soccer players is overrepresented by relatively older players, deriving from the north and centre of the country. Moreover, to the authors’ knowledge, this was the first study that has prospectively explored the future career status of U17 “elite” Italian soccer players, investigating a possible association between birth advantages and the youth-to-senior transition. The prospective statistical analysis conducted in Part 2 of this study revealed how only 3.2% of players were able to develop into a Level 1 player later in their career, while 82% continued playing at the amateur level (50.5%) and/or left any kind of organized soccer practices (31.5%). Furthermore, the findings underlined that players’ ability to successfully develop into Level 1 players was associated with their birthplace, with players born in the north being almost five times more likely to play with a top-tier club at the senior level compared to their counterparts born in the south.

The results from Part 1 of this study demonstrated how Italian players’ participation at the U17 National Championship does not occur on a level playing field, as it is, in most cases, restricted to players born in certain months and regions, with defined chronological ages and geographical areas more easily excluded. The overrepresentation of early born players is due to youth soccer organizations being embedded in a culture of growing professionalization characterized by the continuous assessment of youth players who are valued and ranked based on objective measures of performance [51,52,53,54,55]. Players’ journey within those talent pathways is often defined on their ability to meet performance standards and to fit in with the requirements of the organization [6,7,56,57]. Early born players experience initial benefits consisting of more time to practice, compete, and develop, which eventually provide them greater openings into talent pathways, usually supporting a faster and earlier development of sport-specific skills (i.e., short-term and immediate effects of RAEs [2,58,59] and parents’ initial enrolment bias [60,61]).

On the other side of the same coin, the overrepresentation of players born in Northern and Central Italy at the youth level may be due to the well-known Italian regional disparities, generally labelled using the expression “southern question” [40]. In more detail, a path dependency towards a clientelist style of politics, which characterizes the southern part of Italy, is generally the cause of poor investments from both money misallocation and waste and has subsequently contributed to the north–south dualism (i.e., “southern question”) generally seen today [62], which favours the northern part of the country and that encompasses the entire gamut of civil and economic development indicators (i.e., employment rates, families’ average annual income, individuals’ cultural level, presence, and access to facilities) [63,64]. The “pay-to-play” model that characterizes youth Italian organized soccer, which requires the financial support of parents whereby players’ access to such learning environments is dependent on their families’ sociocultural and economic status, may negatively impact southern players’ future openings to talent pathways. The findings from Part 1 of this study, which have shown a skewed birthplace distribution favouring players from the north and centre, are aligned with previous research conducted in this area and highlight this phenomenon is beyond national team representation and must be framed in the broader Italian regional disparities context.

The results from Part 2 of this study showed RAEs are not a predictor for future career status. Studies have recently underlined that soccer academies are overrepresented by early born players, taller and heavier than their younger selected peers, despite that no differences were found in physical attributes and soccer-specific skills [65,66,67]. This may indicate how differences in physical performance are more associated with the level of competition rather than with relative age [68]. Consequently, early born players’ overrepresentation at the youth level is not due to their talent but due to the immediate and short-term effects of relative age that we have already presented above. It is worth considering here that the few (“lucky”) selected later born players experience the same immediate developmental advantages as the earlier individuals (i.e., access to facilities, better coaches, and competitions); show similar skills and competences [65,66,67]; and in some cases, even possess the greatest likelihood of successfully completing the youth-to-senior transition [69]. This indicates how relative age advantages are a determinant for the early developmental stages but may be overestimated once players are older. Indeed, a recent study conducted on Spanish youth soccer demonstrated how RAEs are not associated with players’ likelihood of accessing the next developmental stage [70]. Moreover, Ginés et al. [71] also showed how, in Spanish male academy soccer, coaches’ selections are not influenced by chronological age, with research conducted by Hill et al. [26] revealing it was not a significant predictor of perceived performance when compared to the biological maturation effect. In line with this, Part 2 of this study highlighted players’ ability to develop into Level 1 players once a senior was not influenced by their relative age, thus indicating early born overrepresentation at the senior level may only be attributed to the knock-on effect (i.e., players selected from a pool of players already affected by RAEs) [72,73]. However, and contrary to what was hypothesized, the results from Part 2 of this study were not able to demonstrate the reversal effect of relative age (i.e., underdog hypothesis), previously revealed across literature on players’ youth-to-senior transition. Thus, showing inconsistencies when exploring RAEs associations with career progressions and success and revealing how different contextual and environmental factors, as well as organizational structures and stakeholders, uniquely influence RAEs outcomes [74].

The results from Part 2 of this study have also highlighted how Italian “elite” youth players’ birthplace is associated with their future career status, whereby players born in the north are almost five times more likely to develop into Level 1 players once they grow senior compared to players born instead in the south. Italian soccer has always been a place where regional disparities are shown [75]. Teams from the north have always been more successful than any other teams across the country, and at the same time, underage successful clubs are mostly located in North and Central Italy, thus facilitating the soccer developmental experiences of players born in those macro-regions who are also favoured in their youth-to-senior transition, likely due to the higher proportion of professional clubs that can be found there (i.e., social visibility) [76]. In contrast, southern players have less opportunity to develop in a higher-performance learning environment and dispose of fewer openings to the next developmental stages. Moreover, teams from the south have historically struggled to maintain their professional and elite status [77,78], indicating how South Italian players develop in a weaker and more uncertain context, which may not favour, and eventually undermine, their transition towards professional soccer; as the results from Part 2 of this study have shown.

However, it is worth noticing how there is a need to contextualize the associations between sporting performance and the communities’ socioeconomic status. Indeed, contrasting results were obtained across different sports and nations [33,36,45,79,80], thus highlighting how different organizational structures, as well as different customs and traditions (i.e., deliberate practice, deliberate play, and free play), that can be found across countries distinctly define interactions with a given community status, shaping the sporting experiences of the youngest generation. For example, in Italian soccer, children and adolescents who want to develop their soccer talent need to be involved in organized soccer practices from the early stages of their developmental journey. It is only through being part of these environments that they can develop early sport-specific skills and dispose of higher social visibility to increase their likelihood of being selected in the future. Indeed, there is no second entrance (or chance) for those not involved in the system early on. Therefore, the pay-to-play model promoted by the Italian soccer federation results in an unintended form of talent wastage in those regions characterized by a lower socioeconomic status. Vice versa, a country such as Brazil, which has been defined by Janet Lever, in her book, as having democratic sporting institutions [81], and in which children and adolescents are exposed to a high level of unstructured play, peer-led activities, and free play, in which they can develop soccer-specific skills outside organized environments [6], may include a second chance for non-early involved children and adolescents. However, different results may also be attributed to the different research designs and methodologies that have been used in birthplace effects studies. 

Despite all selected players having access to the highest developmental opportunities in terms of optimal learning environments (e.g., competent and experienced coaches, access to high-level facilities, and competing and playing with skilled peers), only a few of them will eventually reach the top of the pyramid. One could suggest the presence of a ceiling effect here, whereby not all athletes can achieve the final stage and become professional. Indeed, the strict selection policies and competition pressures that often characterize youth sport can cause the removal of large numbers of players from the sport in question every year [5,52]. However, research conducted into soccer has found players that are recruited at an earlier age by professional club academies do not show a higher probability of attaining a professional contract at the senior level compared to their peers who entered the talent pathway at a later age [21,23]. In line with this, a recent study conducted by Barth et al. [82] showed how youth performance can only explain 2.2% of the variance in senior performance, thus indicating how an increase in the youth performance level does not linearly lead to an increase in future senior performance, suggesting how TID cannot be intended as deterministic [79,82,83,84,85].

Players’ biological maturation, relative age, birthplace, and ethnicity interact together to constrain both players’ developmental opportunities and experiences, thus defining their skill acquisition processes and subsequent performance outcomes. TIDs should then be considered complex social practices [7] that are not only dependent on the microlevel of practice but are rather also being influenced by the social, cultural, economic, and political landscape. Research has highlighted how prediction decisions based solely on current performance outcomes are difficult to make and often unreliable [15]. The prospective analysis conducted in Part 2 of this study contributed to highlight the fallacy of the deterministic assumptions, as, contrary to sport systems’ expectations, 96.8% of “elite” youth players were not able to achieve Level 1 status, and 82% of them did not sign a professional contract later in their career (i.e., 50.5% of “elite” Italian youth players will continue playing at the amateur level, while 31.5% will instead stop organized soccer), thus highlighting the uncertainties around the developmental pathways, as well as the important implications these processes could have on dropouts in the long term (i.e., almost one-third of players have stopped playing altogether).

### 4.1. Practical Implications and Future Directions

Morganti et al. [1], in their recent article, proposed a probabilistic approach to TID, which, based on ecological psychology [86], aims to counteract the deterministic assumption in sporting contexts. More in detail, a probabilistic TID recognizes the intertwined relationship between the self and its environment and thus considers athletes as individuals embedded in an ecology of relations [87]. In this way, their development appears as multifactorial, characterized by uncertainties, which, in turn, makes it difficult to predict senior performance from youth performance. Consequently, to move away from early identification and specialization practices, a probabilistic TID promotes giving sports back to kids and guiding youth sporting activities through an ethos of amateurism. These would encourage shifting the focus in youth sport from performance to participation and from early selection to long-term development, therefore contrasting the proliferation of selection biases due to birth advantages, which research has shown are due to a high level of competition from the very early developmental stages.

This study has underlined how different socioeconomic statuses (North vs. South) interact differently with a given organizational structure (i.e., pay-to-play model), thus alerting against “copy and paste” templates [88], even across the same nation. Specifically, the pay-to-play model promoted by the Italian Soccer Federation resulted in equitable and functional TID practices in North Italy but, in contrast, revealed unfair and less capability of developing new talent in South Italy. As such, future research should direct its focus on the networks of relations that happen between sports organizations and contextual features, investigating the culture and philosophy around talent developmental processes and promoting qualitative lines of inquiry able to investigate both practitioners’ and players’ experiences in sporting environment. This would help sports organizations design and promote TIDs that are equitable and functional across different communities.

Moreover, further research on interactions between the “southern question” and Italian soccer should aim to investigate players’ place of early development and the presence of eventual patterns of migration of players from the south to north and central regions [89,90].

### 4.2. Limitations

When interpreting the results of this study, it is important to consider its limitations. First, only being part of the U17 roster was required to be included in this study. However, some players could have played in considerably more games than others. Youth career duration and/or appearances could be a variable included to understand the influence of birth advantages on long-term development outcomes. Second, this study considered players’ future career status, not taking into consideration their whole career and their senior appearances. Players included in the study were born between 1999 and 2001 and therefore, at the date of the study, were at the beginning of their senior career. Senior career duration and/or appearances could be a variable included to understand the influence of birth advantages on long-term development outcomes in the future. Third, to analyse birthplace effects, this study has only taken into consideration players’ place of birth. However, a player may be born in a particular region of Italy and then moved elsewhere in the country at a younger age. This underlines the importance of analysing players’ place of early development and migratory patterns together with their birthplace. Finally, we derived their sociocultural and economic statuses from census statistics. Another possible method could have been to investigate the sociocultural and economic statuses associated with national representatives. It is, however, worth considering that this evaluation of the “southern question” provides valuable insights on birthplace effects in Italy.

## 5. Conclusions

This was the first study to investigate the influence of birth advantages (i.e., RAEs and birthplace effects) on the selection into national-level underage soccer clubs and their interactions with the youth-to-senior transition. The results from Part 1 of this study revealed that the U17 National Soccer Championship is a half-Italian affair. The results highlighted an underrepresentation of relatively younger players and those born in South Italy. Part 2 of this study aimed to investigate how birth advantages influence selected players’ future career status. The results demonstrated a high dropout rate among former U17 “elite” youth Italian soccer players, with 31.5% no longer involved in organized soccer activities. In line with this, very few of them (3.2%) were able to develop into Level 1 players. Players’ future career status was associated with their macro-regions of birth, with players born in the north having the greatest likelihood of developing into Level 1 players, thus highlighting how North Italian soccer players continue to be favoured by regional disparities that affect Italy from a sociocultural and economic point of view. In contrast, the results from Part 2 also revealed no association between players’ birthdate and their future career status, suggesting that the overrepresentation of early born players at the senior level may only be due to the knock-on effect of their relative age.

## Figures and Tables

**Table 1:** Birth quarters distribution (BQs) of former U17 “elite” Italian soccer players divided per future career status.

Career Status	BQ1 (Expected)	BQ2 (Expected)	BQ3 (Expected)	BQ4 (Expected)	χ2	*p*	V	Effect
Level 1	10 (14.8)	13 (9.9)	7 (6.4)	4 (2.9)				
%	28.4 (43.5)	38.2 (29.1)	20.6 (18.8)	11.8 (8.5)				
Level 2	69 (67.5)	50 (45.2)	25 (29.2)	11 (13.1)				
%	44.5 (43.5)	32.3 (29.2)	16.1 (18.8)	7.1 (8.5)				
Amateur	225 (230.7)	145 (154.5)	112 (99.9)	48 (44.9)				
%	42.5 (43.5)	27.4 (29.2)	21.1 (18.8)	9.1 (8.5)				
Former	153 (144.1)	98 (96.5)	54 (62.4)	26 (28.1)				
%	46.2 (43.5)	29.6 (29.1)	16.3 (18.8)	7.9 (8.5)				
					8.767	0.459	0.91	Very Strong

**Table 2:** Birthplace distribution (macro-regions) of former U17 “elite” Italian soccer players divided per future career status.

Career Status	North (Expected)	Centre (Expected)	South (Expected)	χ2	*p*	V	Effect
Level 1	25 (15)	5 (7.4)	4 (11.7)				
%	73.5 (44)	14.7 (21.7)	11.7 (34.3)				
Level 2	82 (68.3)	32 (33.5)	41 (53.1)				
%	52.9 (44.1)	20.6 (21.6)	26.5 (34.3)				
Amateur	252 (233.7)	104 (114.6)	174 (181.7)				
%	47.5 (44.1)	19.6 (21.6)	32.9 (34.3)				
Former	104 (146)	86 (71.6)	141 (113.5)				
%	31.4 (44.1)	26 (21.6)	42.6 (34.3)				
				42.413	<0.0001	0.142	Moderate

## Data Availability

Data are contained within the article.
